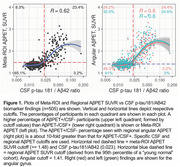# Amyloid PET Detects the Deposition of Brain Aβ Earlier than CSF Fluid Biomarkers

**DOI:** 10.1002/alz.092471

**Published:** 2025-01-09

**Authors:** Val J. Lowe, Carly T. Mester, Emily S. Lundt, Jeyeon Lee, Sujala Ghatamaneni, Alicia Algeciras‐Schimnich, Michelle R Campbell, Jonathan Graff‐Radford, Aivi T. Nguyen, Hoon‐Ki Min, Melissa E. Murray, Matthew L. Senjem, Christopher G. Schwarz, Kejal Kantarci, Brad F. Boeve, Prashanthi Vemuri, David T. Jones, David S. Knopman, Clifford R. Jack, Ronald C. Petersen, Michelle M. Mielke

**Affiliations:** ^1^ Department of Radiology, Mayo Clinic, Rochester, MN USA; ^2^ Mayo Clinic, Rochester, MN USA; ^3^ Hanyang University, Seoul Korea, Republic of (South); ^4^ Department of Neurology, Mayo Clinic, Rochester, MN USA; ^5^ Mayo Clinic, Jacksonville, FL USA; ^6^ Wake Forest University School of Medicine, Winston‐Salem, NC USA

## Abstract

**Background:**

Understanding the relationship between AβPET and AβCSF biomarkers will define their potential utility in Aβ treatment. Few longitudinal or neuropathological comparisons have been reported. We assessed the relationship of AβPET and AβCSF biomarkers in a large community cohort.

**Method:**

Participants 50+ years with coincident AβPET and AβCSF biomarkers (pTau181/Aβ42, n=505, and Aβ42/40, n=54) were included. Of these participants, a subgroup was identified that had neuropathology data (n=47) of which many had longitudinal AβPET and CSF testing. The relationships of AβPET and CSF biomarkers were assessed cross‐sectionally in all participants and longitudinally in those with autopsy data. Analyses were performed using AβPET Meta‐ROI and AβPET early‐Aβ‐deposition ROIs (ROIs with early Aβ deposition: fusiform, angular, inferior temporal, middle temporal, middle occipital, and calcarine).

**Result:**

In biomarker tests performed at the same time, more participants were AβPET+ and CSF‐ than AβPET‐ and CSF+ using Meta‐ROI AβPET. This discordance occurred more frequently when using early‐Aβ‐deposition regions (Figure 1). Of 47 participants with autopsy data, 35 had Thal stage ≥1. The sensitivity for earliest detection of cortical Aβ neuropathology by AβPET was 89% (31/35) and by CSF was 66%, (23/35). Eight of these 35 had positive AβPET findings and negative coincident CSF tests at the first time tested. Of these, 5/8 were Meta‐ROI and early AβPET ROI positive. The other 3/8 were only positive on early‐Aβ AβPET ROIs. Four of the 8 with negative CSF and positive AβPET had subsequent testing and 2/4 became positive on CSF tests after 1.5 years while 2/4 continued to be negative on subsequent testing.

**Conclusion:**

AβPET is more sensitive for detection of early cortical Aβ deposition than CSF biomarkers in cross‐sectional or autopsy data. While the assessment of AβPET by standard Meta‐ROI AβPET methods showed cortical Aβ deposition occurring when not indicated by CSF biomarkers, AβPET ROIs for early Aβ deposition detected additional participants that were AβPET positive and AβCSF negative. These findings have implications for detecting early Aβ neuropathology among CU participants in Aβ prevention trials when robust detection is needed. Further work is needed to compare Aβ plasma biomarkers and AβPET using these methods.